# HIV-associated vaginal microbiome and inflammation predict spontaneous preterm birth in Zambia

**DOI:** 10.1038/s41598-022-12424-w

**Published:** 2022-05-20

**Authors:** Joan T. Price, Bellington Vwalika, Michael France, Jacques Ravel, Bing Ma, Humphrey Mwape, Katelyn J. Rittenhouse, Kristina De Paris, Marcia Hobbs, Julie A. Nelson, Margaret P. Kasaro, Elizabeth M. Stringer, Jeffrey S. A. Stringer

**Affiliations:** 1 Department of Obstetrics and Gynaecology, University of Zambia School of Medicine, Lusaka, Zambia; 2 Department of Obstetrics and Gynecology, University of North Carolina at Chapel Hill, 101 Manning Drive, Chapel Hill, NC 27514 USA; 3 UNC Global Projects–Zambia, Lusaka, Zambia; 4 Institute for Genome Sciences, Department of Immunology and Microbiology, University of Maryland, Baltimore, MD USA; 5 Department of Microbiology and Immunology, University of North Carolina at Chapel Hill, Chapel Hill, NC USA

**Keywords:** Microbial communities, HIV infections, Predictive markers

## Abstract

A *Lactobacillus*-deficient, anaerobe-rich vaginal microbiome has been associated with local inflammation and spontaneous preterm birth (sPTB), but few studies have assessed this association in the setting of HIV. We performed metagenomic sequencing and inflammatory marker assays on vaginal swabs collected in pregnancy. We grouped samples into 7 metagenomic clusters (mgClust) using the non-redundant VIRGO catalogue, and derived inflammatory scores by factor analysis. Of 221 participants, median Shannon diversity index (SDI) was highest in HIV+ with detectable viral load (1.31, IQR: 0.85–1.66; p < 0.001) and HIV+ with undetectable virus (1.17, IQR: 0.51–1.66; p = 0.01) compared to HIV− (0.74, IQR: 0.35–1.26). Inflammatory scores positively correlated with SDI (+ 0.66, 95%CI 0.28, 1.03; p = 0.001), highest among anaerobe-rich mgClust2–mgClust6. HIV was associated with predominance of anaerobe-rich mgClust5 (17% vs. 6%; p = 0.02) and mgClust6 (27% vs. 11%; p = 0.002). Relative abundance of a novel *Gardnerella* metagenomic subspecies > 50% predicted sPTB (RR 2.6; 95%CI: 1.1, 6.4) and was higher in HIV+ (23% vs. 10%; p = 0.001). A novel *Gardnerella* metagenomic subspecies more abundant in women with HIV predicted sPTB. The risk of sPTB among women with HIV may be mediated by the vaginal microbiome and inflammation, suggesting potential targets for prevention.

## Introduction

Extensive research and development efforts have been made to investigate the epidemiological risk factors and biological mechanisms that underlie preterm birth (PTB), to test preventive interventions, and to optimize care for babies born prematurely. However, very few approaches have achieved widespread feasibility and impact, and the burden of PTB has remained high in many regions worldwide^1^. Mothers in sub-Saharan Africa and South Asia face the highest risk of early delivery and often lack access to life-saving neonatal interventions^1^. Maternal HIV infection, which complicates as many as 1 in 4 pregnancies in parts of Africa^2^, can increase the risk of preterm delivery by as much as 50%^3^. Counterintuitively, women with HIV who initiate antiretroviral therapy (ART) before pregnancy may have even higher rates of preterm delivery than those who start ART during pregnancy^4^. The biological mechanism(s) linking the virus and its treatment to PTB remain poorly understood.

PTB is a final common outcome of several distinct pathways that can be broadly delineated by those that follow spontaneous labor or pre-labor membrane rupture and those initiated by a provider due to high-risk maternal or fetal conditions. Many spontaneous PTB (sPTB) phenotypes can be attributed to systemic or local infection, inflammation, or immune activation processes. Few studies to date have characterized the effect of the vaginal microbiome and immune factors on sPTB^5,6^, and limited data exist comparing these factors by maternal HIV serostatus and ART exposure^6–11^. We hypothesize that the elevated risk of preterm delivery faced by women with HIV may be related to measurable differences in the vaginal microbial and immune milieu during pregnancy. In previous analyses, we demonstrated that women with HIV, and particularly those who had not yet started ART at the time of conception, had higher vaginal bacterial diversity and vaginal inflammatory markers^12,13^.

In this follow-up analysis employing whole community metagenomic sequencing and parallel cytokine assays, we investigate whether characteristics of the vaginal microbiome and local immune response are correlated and predict sPTB in women with and without HIV. Metagenomes afford higher resolution characterization of the composition of the vaginal microbiome and clustering both on taxonomic (species) and functional (genes) attributes^14^. Elucidating biological processes by which HIV infection may incite spontaneous preterm labor and delivery could inform the development of effective therapeutic interventions for the prevention of HIV-related prematurity and its consequences.

## Methods

### Study design

The Zambian Preterm Birth Prevention Study (ZAPPS; ClinicalTrials.gov Identifier: NCT02738892) is an ongoing prospective antenatal cohort at the Women and Newborn Hospital of the University Teaching Hospitals (UTH) in Lusaka. Full study procedures have been described previously^15^. Briefly, ZAPPS participants are enrolled prior to 24 gestational weeks and receive comprehensive antenatal care, laboratory testing, biological specimen collection, and ultrasound to establish gestational age. All deliveries prior to 37 gestational weeks were classified as provider-initiated or spontaneous by an on-site obstetrician (JP). sPTB was defined as delivery prior to 37 gestational weeks preceded by spontaneous preterm labor or pre-labor spontaneous rupture of membranes. Preterm labor inductions and pre-labor cesarean deliveries were considered provider-initiated preterm births and excluded from this analysis.

We defined exposures as HIV serostatus at cohort enrollment (HIV− vs. HIV+) and as a 3-level variable that further distinguished HIV+ participants by viral load suppression (i.e., undetectable vs. detectable). We determined HIV infection at enrollment by screening all participants using the SD Bioline 3.0 test (SD Biostandard Diagnostics, India) and confirming positive cases with Determine HIV-1/2 Ag/Ab Combo test (Alere Inc., Waltham, MA). Viral load assays were conducted using the Abbott RealTime HIV-1 Assay (Abbott Molecular, Des Plaines, IL)^16–18^.

### Ethics

The University of Zambia Biomedical Research Ethics Committee and the University of North Carolina Institutional Review Board each granted approval to conduct the ZAPPS study and for protocol-related specimen testing. All research was performed in accordance with relevant guidelines and regulations. Participants in ZAPPS provided individual written informed consent before undergoing study procedures.

### Specimen collection

In ZAPPS, mid-vaginal dry polyester swabs collected at enrollment at subsequent antenatal visits are stored on-site at − 80 °C. Initial specimen selection for vaginal microbiome and cytokine analysis has been explained in detail previously^12,13^. Briefly, to maximize available budget, we used a double sampling technique that selected for participation all eligible HIV+ participants and all HIV− participants who delivered spontaneously prior to 37 gestational weeks. Among HIV− women who delivered at term, we selected participants at random at a proportion determined by available resources. Finally, we also analyzed repeat specimens collected between 24 and 36 gestational weeks from a random subset of HIV+ participants. All staff who performed laboratory analyses were blinded to baseline characteristics and clinical outcomes.

### DNA isolation, sequencing, & bioinformatic analysis

As described in detail previously^12^, we performed whole genome shotgun (WGS) sequencing of bacterial DNA extracted from vaginal swabs. To summarize, genomic DNA was isolated from vaginal swab samples, processed with the Nextera XT DNA Library Preparation Kit (Illumina), purified with Agencourt AMPure XP Reagent, and sequenced on an Illumina HiSeq 4000 system.

Sequencing output from the Illumina HiSeq platform was converted to FASTQ format and demultiplexed using Illumina Bcl2Fastq 2.18.0.12 conversion software (Illumina). Quality control of the demultiplexed sequencing reads were verified with FastQC software (Babraham Institute, Cambridge, UK). Sequencing reads originating from the host were removed using BMTagger (v3.101)^19^ and the human reference genome GRCh38/hg38^20^. The data were then further processed using sortmeRNA (v2.1b) to remove ribosomal RNA sequencing reads^21^ and then Trimmomatic (v0.3653) for quality filtering/trimming^22^. The remaining reads were then mapped to the VIRGO non-redundant gene catalog using Bowtie^23^ (v1.2.2) as described previously^14^. Gene length corrected mapping results from VIRGO were used to establish the taxonomic composition of the vaginal microbiota^24^ (Supplemental Table). Within-species diversity of these microbiota was established for only the four most prevalent bacteria and only included samples with at least 90% of the genes of the taxon’s genome: *L. crispatus* (n = 18), *L. iners* (n = 115), *Gardnerella* (n = 152), and *A. vaginae* (n = 85). Briefly, the patterns of gene content for each taxon were subjected to hierarchical clustering using Bray–Curtis dissimilarity and Ward linkage. For each taxon two clusters were identified each representing a group of genes (i.e., a set of strains) co-occurring frequently in this dataset. These clusters were previously coined metagenomic subspecies^14^. Because only *Gardnerella* metagenomic subspecies demonstrated differences in incidence of sPTB, only this taxon’s metagenomic subspecies were integrated in hierarchical clustering of samples using Bray–Curtis dissimilarity and Ward linkage, where the total abundance of *Gardnerella* in each sample was assigned to either metagenomic subspecies *Gardnerella* type 1 or *Gardnerella* type 2. For samples that did not meet our threshold for *Gardnerella* genome coverage to be included into the metagenomic subspecies analysis, the *Gardnerella* relative abundance was assigned to “*Gardnerella* other”. Considering fluctuating taxonomy within the *Gardnerella* genus and for clarity of comparisons between metagenomic subspecies identified in our analysis and species of other genera, herein we refer to these three *Gardnerella* metagenomic types as “metagenomic subspecies”. Statistical support for seven within-study metagenomic clusters was found using silhouette scores. Species level composition for *Gardnerella* was determined for each sample using VIRGO^25^. Shannon diversity index (SDI), a measure of species richness and evenness (alpha diversity), was calculated for each sample as a sum of each individual species’ proportional abundance multiplied by the natural logarithm of this same proportion^26^.

### Inflammatory marker analysis

A second vaginal swab collected at the same timepoints was analyzed by multiparameter bead array for interleukin-1β (IL-1β), IL-2, IL-6, IL-8, IL-10, IL-12p70, interferon-ɣ (IFN-ɣ), IFN-ɣ-induced protein 10 (IP-10), tumor necrosis factor-α (TNF-α), macrophage inflammatory protein-1α (MIP-1α), MIP-1β, and transforming growth factor-β (TGF-β) by Milliplex (Millipore-Sigma, Minneapolis, MN) and secretory leukocyte protease inhibitor (SLPI) and soluble CD14 (sCD14) by single-analyte ELISA (R&D Systems, Minneapolis, MN). As described in detail previously^13^, we used confirmatory factor analysis to derive inflammatory scores for each sample based on activity of the measured pro-inflammatory markers in vaginal swabs with standardized factor loadings at a significance level of *p* > 0.01 (IL-1β, IL-2, IL-6, IL-8, IL-12p70, IP-10, TNF-α, MIP-1β, and sCD14).

### Statistical analysis

We analyzed baseline demographic data of the sub-study cohort, calculating median and interquartile range (IQR) for each continuous variable, and frequency and percentage for each categorical variable. We compared categorical and continuous variables between primary outcome groups using chi-square and linear regression, applying probability weighting to account for sampling technique.

We described overall and relative taxonomic abundances, species diversity, individual inflammatory biomarkers, and inflammatory scores, quantifying their associations with HIV and viral suppression in unadjusted models. Inverse probability sampling weights were applied to account for unequal sampling technique, with robust standard errors computed by the linear variance estimator^27^.

Correlations between relative abundances of key bacterial taxa and inflammatory markers were estimated using Spearman’s rank-order correlation coefficient (*rho*). A weighted linear regression model was built to estimate the association between individual inflammatory markers and SDI among samples collected between 16–20 gestational weeks, adjusting for HIV and detectable viral load at baseline. All inflammatory markers assayed were included in the initial model and then sequentially removed by stepwise backward elimination.

We calculated median SDI and inflammatory scores of baseline samples in each metagenomic cluster and estimated the relationship of each compared to *L. crispatus*-dominated mgClust7 using weighted linear regression. Similarly, SDI and inflammatory scores were calculated for baseline samples among each *Gardnerella* metagenomic subspecies classification. We reported linear associations of Shannon diversity and vaginal inflammation in samples with *Gardnerella* type 1 and type 2 compared to the “other” *Gardnerella* subtype overall and among subgroups of HIV serostatus.

We identified optimal cutpoints for dichotomizing mean relative abundances of key taxa that maximized the product of sensitivity and specificity to predict the outcome of sPTB using the method proposed by Liu^28^. Associations between vaginal microbiota and inflammation by sPTB were calculated as unadjusted and adjusted prevalence ratios using Poisson regression with robust error variance^29,30^. Because twin gestation and short cervical length (< 2.5 cm) are strong independent predictors of sPTB and rare in our cohort, we excluded women with these risk factors from analyses of this outcome. Multivariable models were adjusted for potential confounding due to maternal age, body mass index (BMI), parity, prior PTB, as well as HIV serostatus (in baseline analyses) or detectable viral load (for matched repeat analyses).

## Results

### Baseline characteristics

**Figure 1:**
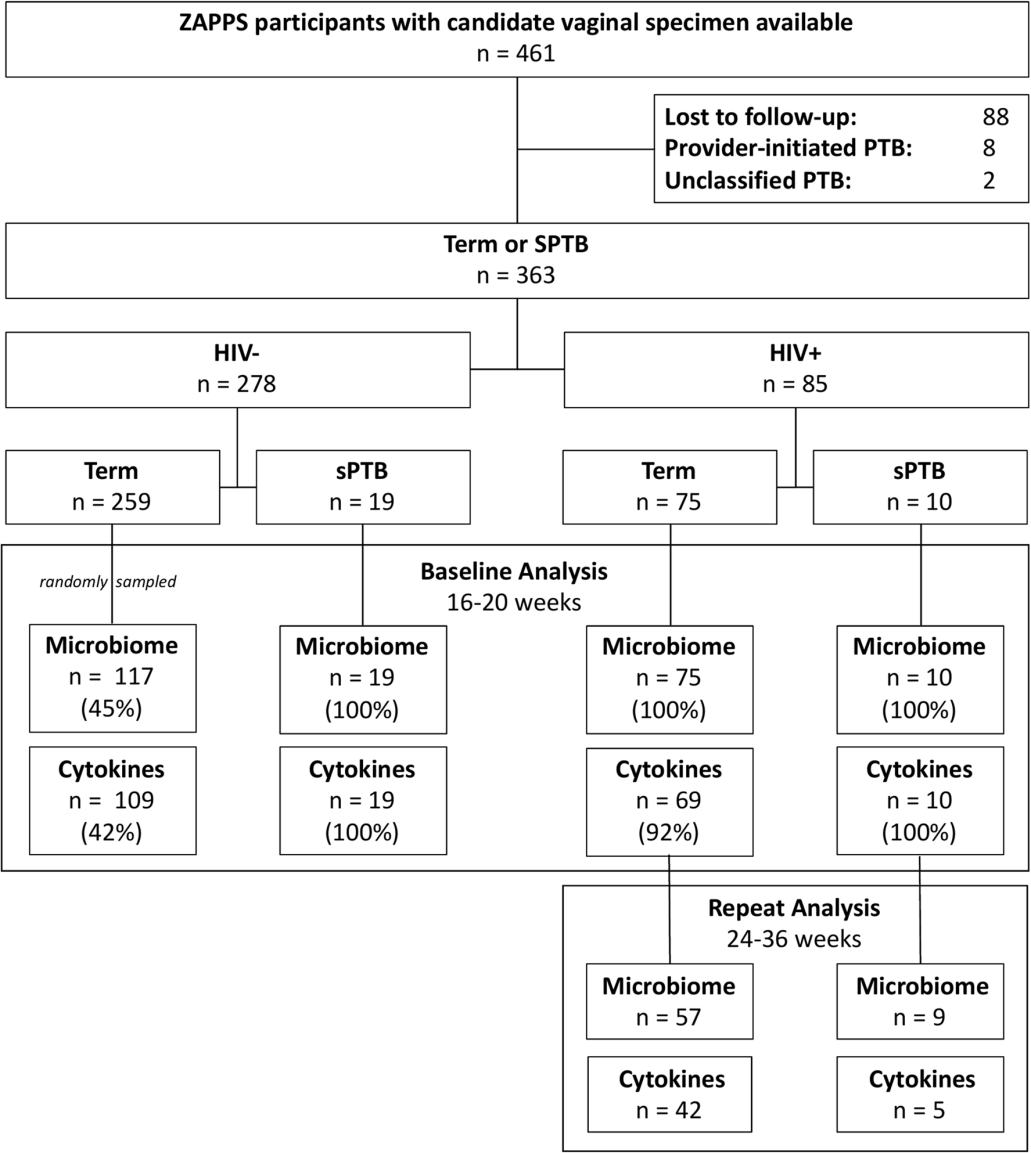
Flowchart of ZAPPS participants with term or spontaneous preterm birth (sPTB) included in vaginal microbiome and cytokine analyses at baseline (16–20 gestational weeks) and repeat (24–36 gestational weeks) timepoints.

Of 221 participants who contributed vaginal specimens to this secondary analysis, 192 (87%) delivered at term and 29 (13%) experienced sPTB (Table 1). Women with sPTB had higher prevalence of prior preterm birth, twin gestation, short cervical length, and vaginal bleeding in pregnancy. Of 85 (38%) participants with HIV at enrollment, 50 (59%) had initiated ART prior to conception, and 39 of those (78%) had undetectable viral load. Median gestational age at first sample collection was 18 weeks (IQR: 17–19). Matched cytokine analysis in baseline samples was available for 207 (94%) participants. We performed repeat microbiome analysis of swabs collected at 32 weeks (IQR: 29–32) among 66 HIV+ participants selected at random, of whom 47 (71%) also underwent a concurrent repeat cytokine analysis (Fig. 1).

**Table 1:** Characteristics of participants with vaginal specimens analyzed in ZAPPS cohort, N = 221.

Baseline characteristic	Term		Spontaneous preterm birth		*p*^*a*^
	N or median	% or IQR	N or median	% or IQR	
Overall^	192	86.9	29	13.2	
**Maternal age—years**	28	(22,32)	27	(24,30)	0.6
< 20	15	8.0	1	3.5	
20–34	148	78.7	25	86.2	
≥ 35	25	13.3	3	10.3	
Missing	4		0		
Married or living with partner	157	82.6	26	89.7	0.3
**BMI—kg/m**^**2**^	24.5	(21.6,27.7)	24.0	(20.6,26.4)	0.2
< 18.5	6	3.1	2	6.9	
18.5–30	150	78.1	24	82.8	
> 30	30	15.6	2	6.9	
**EGA at first specimen collection—weeks**	18	(17,19)	18	(17,19)	0.5
Nulliparous	52	27.1	6	20.7	0.30
Parity	1	(0,2)	2	(1,4)	0.003
Prior preterm birth among parous, N = 163	46	32.9	19	82.6	< .001
Twin gestation	1	0.5	7	24.1	< .001
Cervical length in mid-trimester	3.7	(3.5,4.1)	3.6	(3.2,3.9)	0.02
Vaginal pH	5.5	5.5,6.0	5.5	5.0,6.0	0.3
Sexual intercourse in 24 h prior to sample	148	77.1	20	70.0	0.2
Missing	16		2		
Vaginal washing in 24 h prior to sample	61	31.8	8	27.6	0.6
Missing	176		3		
Syphilis seropositive	23	9.5	0	0	0.2
Missing	8		1		
HIV seropositive	75	39.1	10	34.5	0.1
Preconceptional ART, n = 85	44	60.3	6	60.0	0.9
Missing	2		0		
Viral load undetectable, n = 85	39	52.0	5	50.0	0.9

BMI, body mass index; EGA, estimated gestational age; ART, antiretroviral therapy; IQR, interquartile range.

^a^*p* values calculated compared to term births by χ2 for categorical and linear regression for continuous comparisons, weighted for sampling.

### Taxonomic prevalence, abundances, and metagenomic clustering

A total of 201 unique bacterial species were identified by taxonomic profiling of the metagenomic sequence dataset. The most common bacterial taxa across all 287 specimens collected was *L. iners*, present in all samples (relative abundance IQR: 2%–77%) and all subspecies of the *Gardnerella* genus, present in 99% of samples (relative abundance IQR: 2%–52%). The study-wide mean relative abundance across samples of *L. iners* was 35%, of *Gardnerella* was 30%, of *L. crispatus* was 11%, of *Prevotella* species was 10%, of *A. vaginae* was 4%, and of *Candidatus (Ca.) Lachnocurva vaginae* (formerly BVAB1^31^) was 3%.

**Figure 2:**
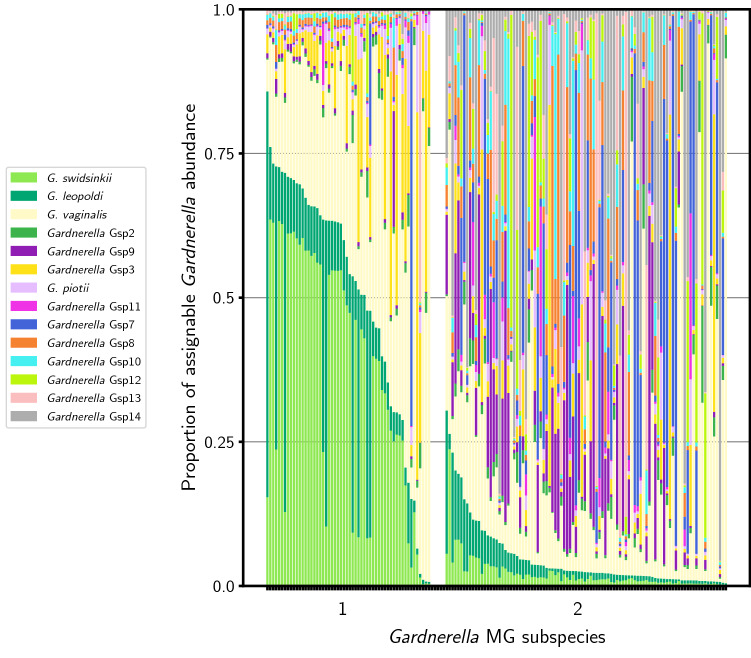
Proportions of assignable *Gardnerella* abundance in two distinct profiles of metagenomic subspecies of *Gardnerella* present in vaginal specimens collected between 16–20 weeks and 24–36 weeks, N = 152.

Two distinct metagenomic subspecies within the *Gardnerella* genus were identified in our samples. The main difference between these metagenomic subspecies involved two recently described, and closely related *Gardnerella* species: *G. swidsinkii* and *G. leopoldii*^25^. The first *Gardnerella* subspecies was characterized by a higher proportion of *G. swidsinkii*/*G. leopoldii* while the second contained a more diverse array of *Gardnerella spp.* and a low proportion of *G. swidsinkii*/*G. leopoldii* (mean proportion of *G. swidsinkii/G. leopoldii* was 48% vs 4%, respectively; Fig. 2). A third profile contained a lower overall relative abundance of *Gardnerella* such that it precluded assignment to a metagenomic subspecies. We refer to these metagenomic subspecies as *Gardnerella* types 1, type 2, and “other”, respectively.

Median SDI across all 287 samples was 0.93 (IQR: 0.47–1.50) and among baseline samples was 0.86 (IQR: 0.44–1.51). Among matched samples from 66 HIV+ participants who also had a later sample analyzed, SDI was similar between baseline (median 1.20, IQR: 0.59–1.65) and repeat (median 1.05, IQR: 0.64–1.49; *p* = 0.7) specimen collection timepoints.

**Figure 3:**
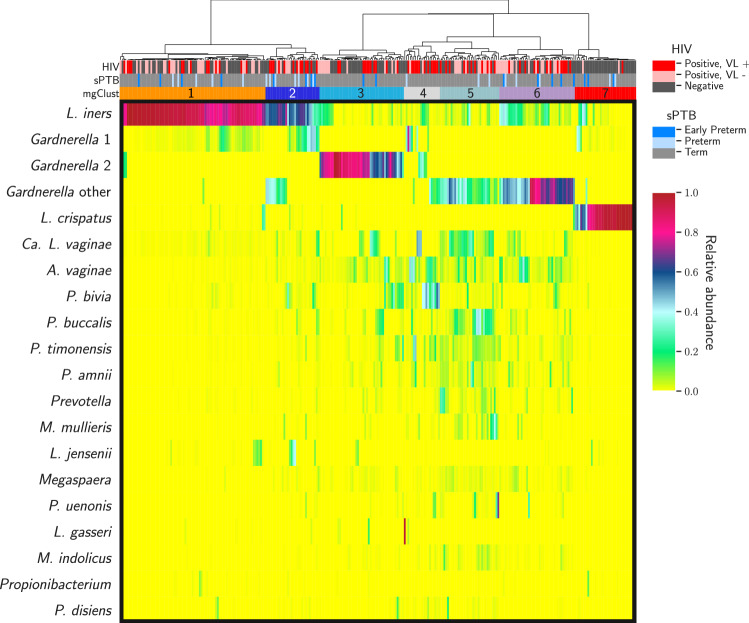
Heat map of vaginal microbial composition of all specimens collected between 16–20 weeks 24–36 weeks, N = 287.

Based on clustering of all samples, 7 major metagenomic clusters (mgClust) were identified (Fig. 3): mgClust1 was dominated by *L. iners* (mean relative abundance 89%); mgClust2 by a mix of *L. iners* (54%) and *Gardnerella “*other” (26%); mgClust3 by *Gardnerella* type 1 (70%); mgClust4 by *P. bivia*, *A. vaginae,* and *Gardnerella* (a mix of all three metagenomic subspecies)*,* mgClust5 by *L. iners, Gardnerella* type 2*,* and *Ca. Lachnocurva vaginae*; and mgClust6 by *Gardnerella* type 2, *L. iners*, and *P. bivia*. Finally, mgClust7 comprised predominantly *L. crispatus* (86%) with minor co-occurrence of other lactobacilli*.*

### Microbiome characteristics by HIV status

Presence of the *Gardnerella “*other” metagenomic subspecies was most common in baseline samples collected from women without HIV (n = 77/136, 57%), intermediate in those collected from HIV+ women with undetectable virus (n = 16/44, 36%; p = 0.03), and least common among those collected from women with detectable virus (n = 10/41, 24%; p = 0.001). Conversely, the presence of *Gardnerella* type 2 was most common in baseline samples from women without HIV (n = 30/136, 22%), intermediate in those collected from HIV+ women with undetectable HIV (22/44, 50%; p = 0.001), and least common among those collected from women with detectable virus (n = 24/41, 59%; p < 0.001).

**Figure 4:**
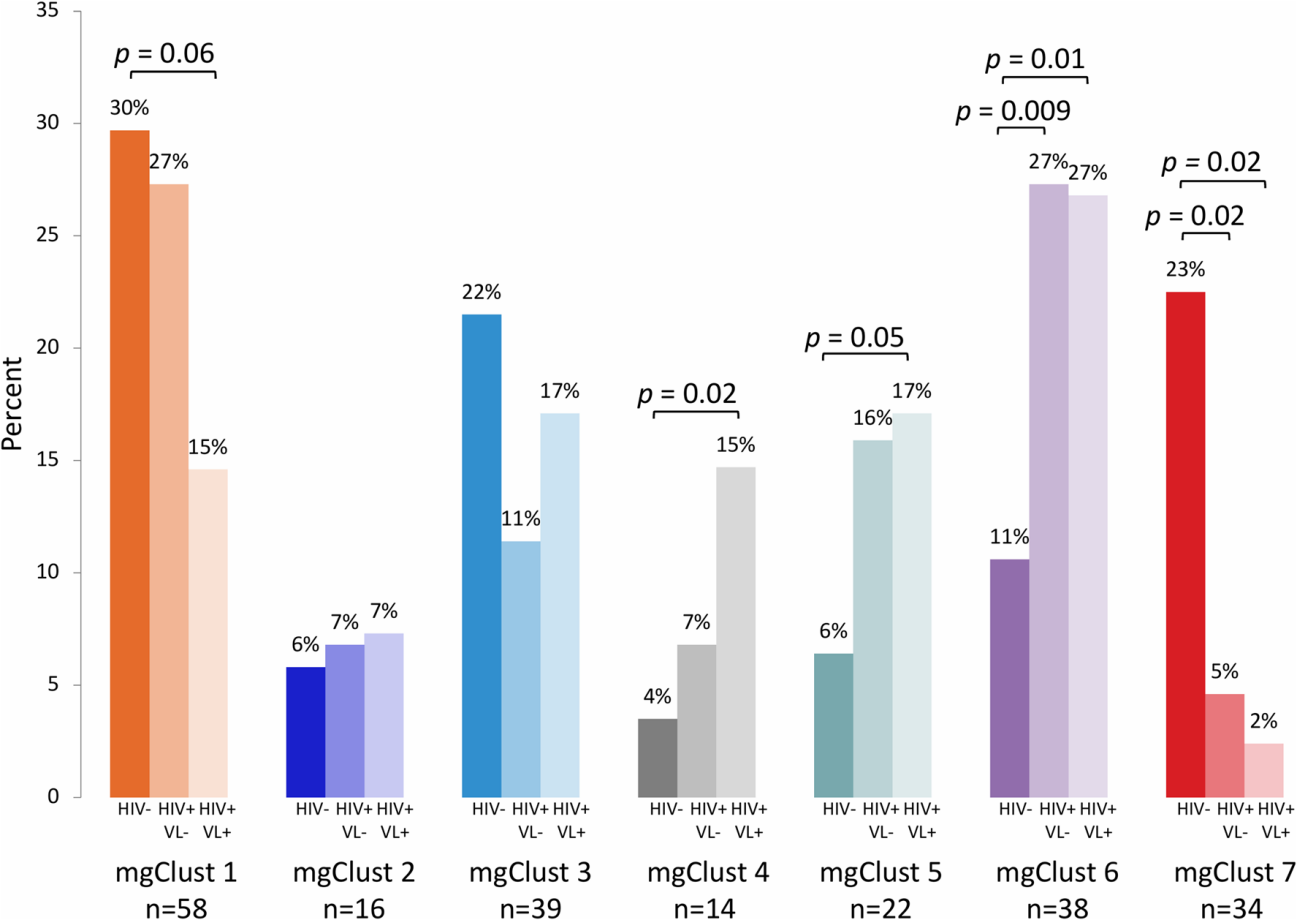
Prevalence of metagenomic community cluster (mgClust) by HIV status and viral load (VL) at 16–20 gestational weeks. Relative percents weighted for sampling and *p* values calculated by weighted logistic regression with HIV− as comparator group.

The relative distribution of vaginal specimens across metagenomic clusters at baseline also differed by HIV serostatus and viral load (Fig. 4). Compared to participants without HIV, women with HIV overall had higher prevalence of microbiota dominated by *Gardnerella* type 2 and other mixed anaerobes in mgClust5 (17% vs. 6%; p = 0.02) and mgClust6 (27% vs. 11%; p = 0.002), and markedly lower prevalence of the *L. crispatus*-dominant mgClust7 (4% vs. 23%; p = 0.001). While women with HIV had modestly higher prevalence of mgClust4 compared to those without HIV (11% vs. 4%; p = 0.05), this was driven by the subset of HIV+ participants with detectable virus who had substantially higher relative prevalence of mgClust4 (15%; p = 0.02). Consistent with our previous results, median SDI at baseline was higher in specimens collected from HIV+ women with undetectable viral load (1.17, IQR: 0.51–1.66; p = 0.01) and highest in those with detectable virus (median 1.31, IQR: 0.85–1.66; p < 0.001) compared to those without HIV (0.74, IQR: 0.35–1.26). Baseline vaginal inflammation scores were statistically similar between HIV+ (median: 0.82, IQR: − 0.39–1.08) compared to HIV− participants (median: 0.40, IQR: − 0.74–1.02; p = 0.3).

### Vaginal inflammation and microbiome characteristics

In vaginal specimens collected at 16–20 weeks, moderate positive correlations were noted between log-transformed concentrations of IL-1β, IL-10, and sCD14 and relative abundances of *Gardnerella* type 2, *A. vaginae*, and *P. bivia*, while negative correlations were found with *L. crispatus* and *Gardnerella “*other*”* (Table 2). Conversely, a moderate negative correlation existed between SLPI and relative abundance of *Gardnerella* type 2 and *A. vaginae*, and positive correlations with *Gardnerella* “other” and *L. crispatus*.

**Table 2:** Correlation between key relative bacterial abundances and log cytokine concentrations, represented as Spearman rank-order coefficients (*rho*). Bolded coefficients represent moderate correlation (i.e., |.3| to |.5|) between bacterial abundance and cytokine concentration and with p < 0.001, adjusted for multiple comparisons by Bonferroni correction.

		Log cytokine concentration							
		IL-1β	IL-2	IL-6	IL-10	IL-12p70	SLPI	sCD14	
		*r*	*r*	*r*	*r*	*r*	*r*	*r*	
Relative bacterial abundance	*L. iners*	− 0.12	0.02	0.11	− 0.13	0.10	0.10	− 0.06	
	*Gardnerella spp.*	**0.38**	0.16	0.07	**0.34**	0.01	− 0.26	**0.42**	
	*G. “other”*	− **0.39**	− 0.22	0.15	− 0.28	− 0.03	**0.30**	− **0.44**	**1.0**
	*G. 1*	0.15	0.00	0.02	0.07	− 0.14	− 0.01	0.17	**0.5**
	*G. 2*	**0.35**	0.24	− 0.08	**0.32**	0.16	− **0.35**	**0.38**	**0.0**
	*L. crispatus*	− **0.38**	− 0.18	0.09	− **0.38**	− 0.05	**0.38**	− **0.45**	− **0.5**
	*BVAB1*	0.15	0.01	0.06	0.18	− 0.07	− 0.01	0.12	− **1.0**
	*A. vaginae*	**0.50**	0.18	0.00	**0.40**	0.09	− **0.33**	**0.45**	
	*P. bivia*	**0.34**	0.24	0.06	**0.40**	0.21	− 0.25	**0.32**	

**Figure 5:**
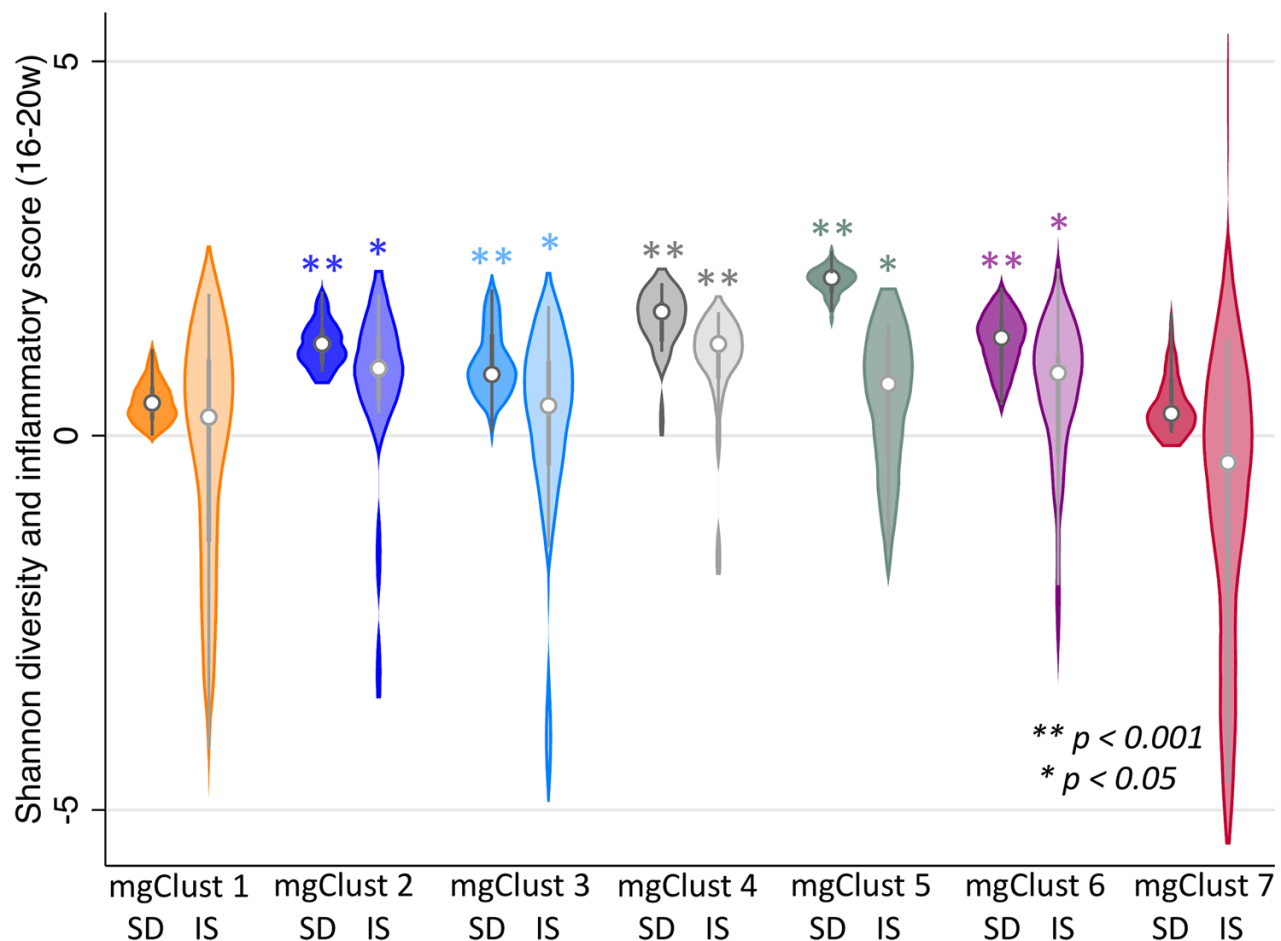
Median Shannon diversity indices (SD) and inflammatory scores (IS) by metagenomic clusters (mgClust) in vaginal specimens collected between 16–20 gestational weeks. *p* values calculated by weighted linear regression of SD and IS in each mgClust compared to *L. crispatus*-dominant mgClust 7, adjusting for HIV serostatus at enrollment.

IL-1β, IL-10, and sCD14 concentrations were associated with higher SDI, while IL-2, IL-6, IL12p70, and SLPI were each modestly associated with lower SDI (Table 3). Inflammatory scores increased with SDI (coeff+ 0.66, 95%CI 0.28, 1.03; p = 0.001); both were highest among specimens in mgClust2, mgClust4, mgClust5, and mgClust6 (Fig. 5), and were moderately correlated overall (*rho* + 0.3; p < 0.001). Compared to *L. crispatus-*dominated mgClust7, both SDI and inflammatory scores were significantly higher in specimens of anaerobe-abundant mgClust2 through mgClust6, but only modestly higher in specimens of *L. iners*-dominated mgClust1 (Table 4). Both SDI and inflammatory scores were lowest in samples with *Gardnerella “*other” and significantly higher in those with *Gardnerella* type 2 (Table 5). The highest SDI and inflammatory scores were noted in samples collected from participants with *Gardnerella* type 2, regardless of HIV status.

**Table 3:** Shannon Diversity Index by log vaginal cytokine concentrations at 16–20 gestational weeks, N = 206.

Cytokine	coeff	95% CI	adj coeff	95% CI	p*
IL-1β	0.13	0.09, 0.17	0.11	0.05, 0.18	0.001
MIP-1α	0.16	0.08, 0.24			
IL-2	0.11	0.02, 0.20	− 0.11	− 0.23, 0.01	0.06
IP-10	− 0.04	− 0.09, 0.01			
IL-6	0.00	− 0.04, 0.04	− 0.03	− 0.07, − 0.01	0.09
IL-8	0.04	− 0.02, 0.10			
IL-10	0.23	0.15, 0.32	0.25	0.15, 0.36	< .001
IFN-γ	0.01	− 0.05, 0.07			
TNF-α	0.13	0.06, 0.21			
MIP-1β	0.21	0.12, 0.30			
IL-12p70	0.04	− 0.04, 0.12	− 0.19	− 0.28, − 0.10	< .001
SLPI	− 0.13	− 0.17, − 0.08	− 0.06	− 0.11, − 0.01	0.02
TGF-β	0.06	0.04, 0.08			
sCD14	0.14	0.10, 0.18	0.06	0.00, 0.11	0.05
**HIV and VL**					
HIV-	1		1		
HIV+, undetectable VL	0.26	0.05, 0.47	0.13	− 0.05, 0.32	0.2
HIV+, detectable VL	0.42	0.20, 0.63	0.22	0.03, 0.40	0.02

Coefficients, 95% confidence intervals, and associated *p* values calculated by linear regression.

**Table 4:** Shannon diversity indices and inflammatory scores by metagenomic cluster (mgClust) at 16–20 gestational weeks.

	Shannon Diversity Index					Inflammatory Score				
	Median	IQR	coeff	95% CI	*p*	Median	IQR	coeff	95% CI	*p*
mgClust 1	0.44	0.22–0.65	0.03	− 0.14, 0.20	0.7	0.25	− 1.42–1.02	0.61	− 0.29,1.52	0.2
mgClust 2	1.23	0.96–1.44	0.78	0.56, 0.99	< .001	0.59	0.48–1.32	1.66	0.71,2.60	0.001
mgClust 3	0.82	0.70–1.35	0.51	0.29, 0.73	< .001	0.05	− 0.40–1.00	0.97	0.05,1.88	0.04
mgClust 4	1.66	1.26–1.79	1.01	0.62, 1.39	< .001	0.91	0.76–1.35	1.95	0.98,2.91	< .001
mgClust 5	2.11	1.91–2.17	1.59	1.40, 1.78	< .001	0.42	− 0.30–1.29	1.45	0.47,2.43	0.004
mgClust 6	1.31	0.95–1.55	0.81	0.57, 1.05	< .001	0.44	− 0.23–1.08	1.41	0.47,2.35	0.003
mgClust 7	0.30	0.11–0.76	*ref*	–	–	− 0.75	− 2.00–0.47	*ref*	–	–

Coefficients, 95% confidence intervals, and associated *p* values calculated by weighted linear regression, adjusting for HIV serostatus at enrollment.

**Table 5:** Shannon diversity indices and inflammatory scores by *Gardnerella* metagenomic subspecies at baseline at 16–20 gestational weeks.

	Shannon Diversity Index					Inflammatory Score				
	Median	IQR	coeff	95% CI	*p*	Median	IQR	coeff	95% CI	*p*
*Gardnerella*—“other”	0.44	0.21–0.85	*ref*	–	–	0.14	− 1.49 to 0.96	*ref*	–	–
HIV− (n = 77)	0.44	0.20–0.81	*ref*	–	–	0.03	− 1.33 to 0.93	*ref*	−	–
HIV+ (n = 26)	0.39	0.22 to 0.88	*ref*	–	–	0.21	− 2.31 to 1.02	*ref*	–	–
*Gardnerella*—1	0.82	0.70–1.35	0.43	0.26,0.60	< .001	0.51	− 0.33 to 1.06	0.53	− 0.06,1.12	0.08
HIV− (n = 29)	0.79	0.69–1.35	0.42	0.23,0.62	< .001	0.51	− 0.23 to 1.07	0.51	− 0.17,1.18	0.1
HIV+ (n = 13)	1.06	0.76–1.26	0.46	0.15,0.78	0.005	0.53	− 0.57 to 1.04	0.70	− 0.40,1.81	0.2
*Gardnerella*—2	1.54	1.10–1.94	0.95	0.78,1.12	< .001	0.91	0.13–1.29	0.92	0.44,1.41	< .001
HIV− (n = 30)	1.53	1.03–1.96	0.97	0.76,1.19	< .001	0.83	0.03–1.30	0.78	0.16,1.39	0.01
HIV+ (n = 46)	1.54	1.19–1.91	0.91	0.65,1.16	< .001	0.93	0.35–1.29	1.27	0.49,2.05	0.002

Coefficients, 95% confidence intervals, and associated *p* values calculated by weighted linear regression.

### Effect of microbiome and inflammation on sPTB

In empirical analysis of optimal cutpoints among baseline samples, mean relative abundance of *L. iners* above 26% (prevalence ratio, PR 2.5; 95% CI: 1.2, 5.2; p = 0.02), *Gardnerella “*other*”* above 0.3% (PR 2.5; 95% CI 1.2, 5.4; p = 0.02), and *Gardnerella* type 2 above 50% (PR 2.6; 95% CI 1.1–6.4; p = 0.03) were each associated with sPTB and remained so in models adjusted for HIV and viral load. Whereas mean relative abundances of *L. iners* and *Gardnerella “*other*”* among baseline samples were similar by maternal HIV status, compared to samples collected from participants without HIV (10%), women with HIV had higher mean relative abundance of *Gardnerella* type 2 (23%; p = 0.001). Although no cutpoint of *Gardnerella* type 1 was found to protect against sPTB, mean relative abundances were higher in samples from women who delivered at term (14%) compared to sPTB (3%; p < 0.001) and trended higher among women without HIV (15%) compared to those with HIV (10%; p = 0.08). Finally, mean relative abundances of *L. crispatus* were similar between samples from term (13%) compared to sPTB (15%; p = 0.9) but were lower among women with HIV (3%) compared to those without (20%; p < 0.001).

**Figure 6:**
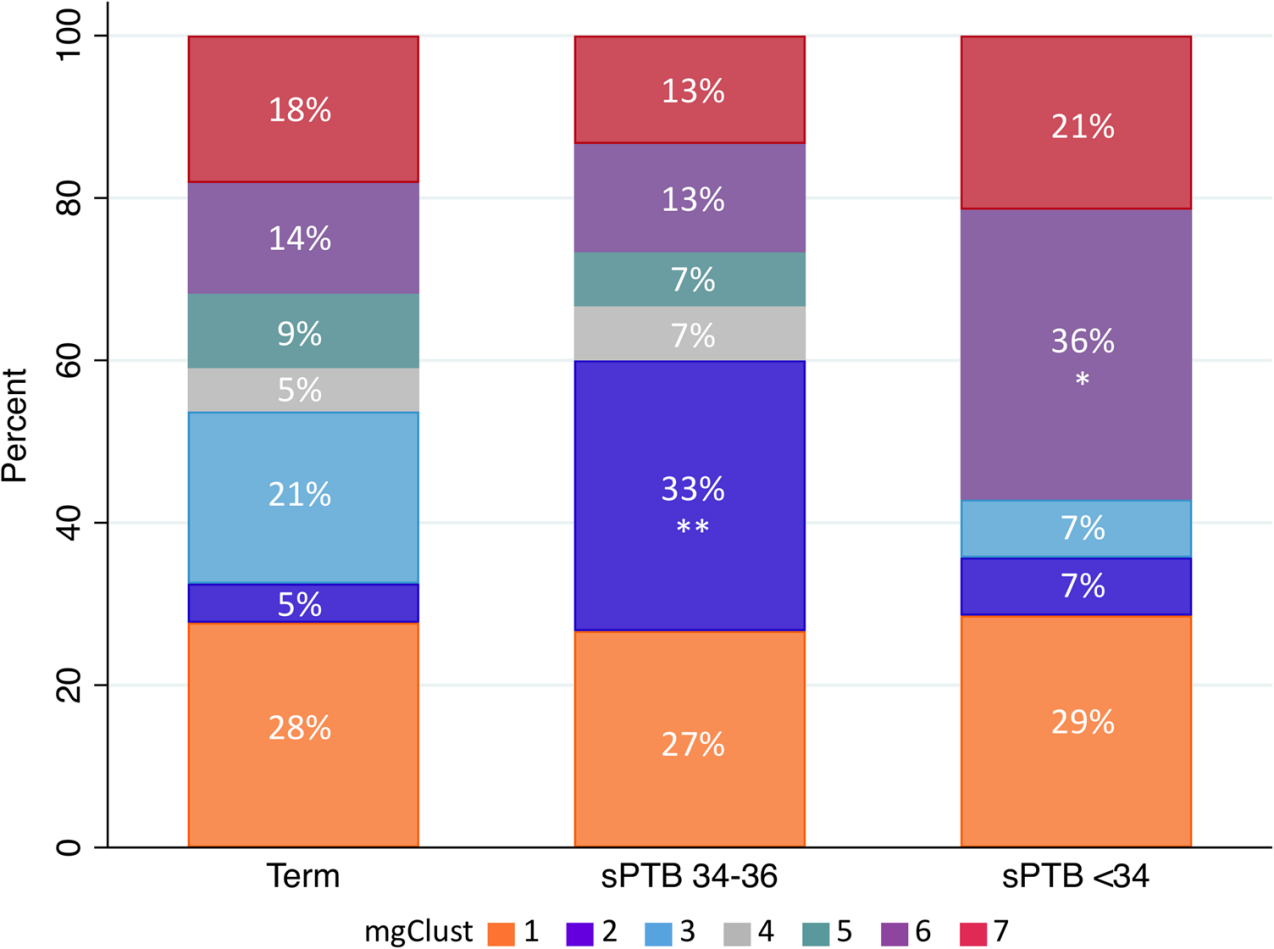
Prevalence of metagenomic clusters (mgClust) at 16–20 gestational weeks among participants with term birth, spontaneous preterm birth at 34–36 weeks (sPTB 34–36), and spontaneous preterm birth before 34 weeks (sPTB < 34). Relative percents weighted for sampling and *p* values calculated by weighted Poisson regression of prevalence of mgClust between preterm birth outcomes compared to term; * *p* < 0.05; ** *p* < 0.001.

Among 192 participants who delivered at term, 10 (5%) had baseline specimens in mgClust2 compared to 5 (33%) among 16 who had sPTB between 34–36 weeks (PR 6.9; 95% CI 2.6, 18.3; p < 0.001) (Fig. 6). When restricting the outcome to sPTB < 34 weeks, 14 (6%) participants met this more severe outcome, of whom 5 (36%) had baseline specimens in mgClust6 (PR 2.6; 95% CI 1.2, 5.8; p = 0.02).

Baseline vaginal inflammatory scores were higher among participants who experienced sPTB (median 0.90, IQR: 0.03–1.28) compared to those who delivered at term (median 0.50, IQR: − 0.73–1.04) (Table 6). The prevalence of sPTB increased with higher vaginal inflammatory scores at baseline in models weighted for sampling and adjusted for maternal age, BMI, parity, prior PTB, and HIV serostatus (APR 2.8, 95% CI: 1.5, 5.2; p = 0.001). Baseline SDI were similar between those who experienced sPTB and those who delivered at term.

**Table 6:** Shannon diversity index and vaginal inflammatory scores at baseline (16–24 weeks) and the change (Δ) from baseline to repeat (24–36 gestational weeks) between participants experiencing term birth and spontaneous preterm birth (sPTB).

	Term median (IQR)	sPTB median (IQR)	PR (95% CI)	APR (95% CI)	*p*
**Baseline analysis**					
Shannon diversity index, n = 221	0.82 (0.43, 1.55)	0.92 (0.59, 1.26)	1.3 (0.7, 2.3)	1.4 (0.6, 3.5)	0.4
Vaginal inflammatory score, n = 207	0.50 (− 0.73, 1.04)	0.90 (0.03, 1.28)	1.4 (1.0, 1.9)	2.8 (1.5, 5.2)	0.001
**Repeat analysis among participants with HIV**					
Δ Shannon diversity index, n = 66	0.11 (− 0.54, 0.37)	0.18 (0.11, 0.21)	2.1 (1.1, 3.8)	2.5 (1.1, 5.6)	0.03
Δ Vaginal inflammatory score, n = 47	− 0.11 (− 0.81, 0.72)	0.37 (− 0.07, 0.48)	1.1 (0.8, 1.5)	1.3 (0.7, 2.2)	0.4

Multivariable model estimates of the prevalence of spontaneous preterm birth (APR) calculated by weighted Poisson regression with robust error variance and adjusted for HIV serostatus (in baseline models) or detectable viral load (in change models), maternal age, body mass index, parity, and prior PTB.

### Microbial stability and inflammatory changes among HIV+ women

Among 66 participants with HIV who contributed vaginal specimens for vaginal microbiome characterization at a second timepoint (median 32 weeks; IQR: 29–32), 32 (48%) had undetectable viral load at enrollment and had initiated ART before pregnancy. Of the 34 (52%) with detectable virus, 5 (15%) had started ART before pregnancy. Between baseline specimens collected at 16–20 weeks and repeat specimens, median change in SDI was statistically similar among the 37 participants who had initiated ART before pregnancy (+ 0.11; IQR: − 0.28–0.48) compared to the 29 who had not (− 0.17; IQR: − 0.41–0.25; rank sum p = 0.5). Vaginal inflammation overall decreased in women who had started ART before pregnancy (median -0.33, IQR: − 0.97–0.34) while it increased in participants who had not (median 0.31, IQR: − 0.22–0.90; rank sum p = 0.02).

Nine (14%) participants with repeat vaginal samples analyzed delivered spontaneously before term. SDI increased between baseline and repeat collection timepoints among women who went on to have a sPTB (median + 0.18, IQR: 0.11–0.21) while it decreased among those who delivered at term (median − 0.11, IQR: − 0.54–0.37). In adjusted models, increasing SDI predicted sPTB (APR 2.5; 95%CI 1.1, 5.6). Vaginal inflammatory scores increased between baseline and repeat specimens modestly more among participants who experienced sPTB (median + 0.37, IQR: − 0.07–0.48) compared to those who delivered at term (median − 0.11; IQR: − 0.81–0.72), but confidence intervals were wide and included the null in weighted multivariable models.

## Discussion

We employed metagenomic sequencing of the vaginal microbiome and assays of local inflammatory markers to investigate the relationships between the microbiome and sPTB in a cohort of pregnant women with and without HIV in Zambia. Our analysis confirms a high prevalence of diverse, anaerobe-rich microbiota in our cohort overall, and particularly among women with HIV. In this analysis, vaginal samples were classified into 7 distinct types based on clustering metagenomic content, whose distribution varied by maternal HIV and, in some cases, by viral suppression status. Two groups dominated by *Gardnerella* and *L. iners* species predicted sPTB. We identified two *Gardnerella* metagenomic subspecies (a group of co-occurring strains/species of *Gardnerella* defined by gene content) whose prevalence varied by maternal HIV serostatus and viral suppression, were associated with varying levels of microbial diversity and vaginal inflammation, and differentially predicted sPTB. These *Gardnerella* metagenomic subspecies were distinguished by the proportion of *G. swidsinkii*/*G. leopoldii*, two closely related *Gardnerella* subspecies^25^. *Gardnerella* metagenomic subspecies type 1, dominated by *G. swidsinkii*/*G. leopoldii,* was modestly less abundant both in women with HIV and those who delivered sPTB. In contrast, *Gardnerella* metagenomic subspecies type 2, notable for a highly diverse mix of other subspecies of *Gardnerella*, was more abundant among women with HIV and those who delivered sPTB. Vaginal microbiota dominated by *L. crispatus,* although very uncommon among women with HIV and rare overall, did not confer the anticipated protective effect against preterm birth as presented in other cohorts^32^. Finally, we described multiple correlations between vaginal microbiome and inflammatory markers and found that both vaginal inflammation among all participants at baseline and an increase in microbial diversity through pregnancy among a subset with HIV were associated with sPTB.

Similar to other studies during and outside of pregnancy, pregnant women with HIV in ZAPPS exhibited more diverse and anaerobe-rich vaginal microbiota compared to women without HIV. However, a preponderance of literature in sub-Saharan Africa demonstrates that it is *Lactobacillus*-deficient, anaerobe-rich vaginal microbiota that confers a higher susceptibility to HIV infection itself, such that it remains unclear whether HIV is a cause or effect of increased microbial diversity and vaginal inflammation in pregnancy. The uncertain causal relationship between HIV infection and the vaginal microbiome is further complicated by broader shifts towards *Lactobacillus* dominance mediated by a physiologic estrogen excess during pregnancy, which may be disrupted by HIV-related chronic inflammation, immune reactivation with ART initiation, or certain antiretroviral agents. Although our analysis was limited in size, women who had started ART prior to conception had a reduction in vaginal inflammation throughout pregnancy compared to those who had not^13^, while the change in alpha diversity (SDI) trended in the opposite direction. This may indicate that ART initiation activates local inflammatory pathways with either minimal or modest benefit on the microbial milieu, but longitudinal analyses of vaginal microbiota from preconception through pregnancy, and from pre- and post-ART initiation, are needed to confirm this hypothesis. Furthermore, whereas nearly all participants with HIV in ZAPPS were taking efavirenz-based regimens, dolutegravir-based ART is now recommended as first line instead such that future research will need to address any differential effects between these exposures.

In previous reports derived from the same ZAPPS cohort, we described associations between HIV and sPTB^33^, HIV and anaerobe-rich microbiota^12^, ART initiation and vaginal inflammation^13^, and vaginal inflammation among women who experienced sPTB^13^. In this analysis that employed updated metagenomic sequencing, linked the vaginal microbiome to inflammation, and analyzed associations between the microbiome and birth outcomes, we found correlations between vaginal inflammatory markers and microbiome and demonstrated that metagenomic characteristics more common among women with HIV were associated with inflammation and sPTB. Using 16S rRNA gene sequencing to characterize the vaginal microbiota, Gudza-Mugabe and colleagues reported that, whereas pregnant women living with HIV in Zimbabwe had higher prevalence of diverse, anaerobe-rich microbiota, and moderate correlations were noted between certain bacterial taxa and vaginal cytokines, no association was found between HIV and inflammation, and the higher risk of preterm birth among women with HIV was independent of the vaginal microbiota. In contrast, we found characteristics of the vaginal microbiome and inflammation predicted sPTB even after adjusting for HIV serostatus and viral suppression. Methodological differences may limit direct comparison between the Gudza-Mugabe report and our study, including methods of estimating gestational age, gestational ages at assessment, distinctions in viral load suppression and ART initiation timing, and the higher resolution in *Gardnerella* speciation by metagenomic sequencing. Furthermore, HIV differentially increases the risk of spontaneous over provider-initiated preterm deliveries such that risk estimates and associations may be blunted when examining PTB overall^33^; this may partly explain the null results reported by others.

The direct causes of sPTB are often unknown, but overt infection leading to inflammation and immune activation are common antecedents. In concert with findings among pregnant and non-pregnant women^6,34–37^, we found moderate correlations between vaginal inflammation and the composition of the vaginal microbiome at baseline, and independent associations between the vaginal microbiome and risk of sPTB. However, over half of our participants had anaerobe-rich *Lactobacillus*-deficient type of vaginal microbiota and a similar proportion experienced sPTB as those with microbiota dominated by *L. crispatus*, commonly considered protective and anti-inflammatory. Although we noted a trend in *L. iners*-dominant communities found more commonly in women with late sPTB between 34 and 36 weeks compared to *G. vaginalis-*rich communities in women with earlier sPTB < 34 weeks, additional studies with larger sample sizes are needed to confirm whether vaginal microbiome composition differentially predicts severity of prematurity. Furthermore, clear geographic variations in common vaginal microbial characteristics and the associated risk of preterm birth highlight the need for population-specific approaches to classifying the vaginal microbiome and to identifying women at highest risk who will most benefit from preventive therapies^5,7,38–41^. In our population, relative abundance of metagenomic subspecies of *Gardnerella* may better convey risk or protection than uncommon *Lactobacillus* species or community state type classifications. Further examination of the functional make-up of *L. crispatus* in this cohort and others where it was found protective is warranted.

We acknowledge several limitations to this analysis. Because of the nature of our observational cohort that relied on standard antenatal care practices, we could not investigate the role of sexually transmitted infections other than HIV and syphilis and we were limited by some missingness in baseline covariates. Additionally, we did not collect data on recent antibiotic use, which could differ by HIV serostatus but would likely bias our findings toward no association; nonetheless, recent antibiotic use was conceivably uncommon in the baseline cohort of samples collected at the first antenatal visit. Due to our sample selection procedure, the characteristics of this nested study are not directly generalizable to the full cohort. Although we weighted analyses to account for sample selection using inverse probabilities, we are currently undertaking more comprehensive analyses in a much larger sample of our cohort population to better characterize the interactions between HIV and ART, the vaginal microbiome and inflammation, and adverse birth outcomes. Similarly, due to limited funding, the current analyses were unable to investigate longitudinal differences between participants with and without HIV. The use of metagenomic sequencing is a strength in this study and afforded a higher resolution of *Gardnerella* which 16S rRNA gene sequencing cannot provide.

In summary, pregnant women in Zambia have high prevalence of anaerobe-rich vaginal microbiota correlated with local inflammation, and women with HIV exhibit characteristics of the vaginal microbiome associated with spontaneous preterm birth. These findings suggest the risk of preterm birth faced by women with HIV may be mediated by the vaginal microbial and inflammatory environment and could be a target for novel preventive therapies aimed at restoring a protective vaginal microenvironment. However, since many women with diverse vaginal microbiota and inflammation still deliver at term and certain species and subspecies differentially confer risk across populations, additional research is needed to identify women who would most benefit from intervention, to define how risk is modified by other host factors, and to tailor interventions to population and individual risk profiles.

## Supplementary Information

Supplementary Information.

## Data Availability

Individual de-identified participant data that underlie the results reported in this article are publicly available at Open Science Framework (https://doi.org/10.17605/OSF.IO/WT6Q8). Sequence read data have been deposited in the Sequence Read Archive (SRA) of the US National Institutes of Health (submission ID: SUB10306692).
